# Functional Postoperative Outcome for 92 Cases of Radial Head Fractures: A PROM-Based Retrospective Study

**DOI:** 10.3390/jcm12185870

**Published:** 2023-09-09

**Authors:** Michael Müller, Verena Mann, Julian Zapf, Konstantin Kirchhoff, Michael Zyskowski, Peter Biberthaler, Chlodwig Kirchhoff, Markus Wurm

**Affiliations:** Department of Trauma Surgery, Hospital Rechts der Isar, Technical University of Munich, 81675 Munich, Germanyjulian.zapf@mri.tum.de (J.Z.); michael.zyskowski@mri.tum.de (M.Z.); markus.wurm@mri.tum.de (M.W.)

**Keywords:** proximal radius, patient-reported outcome, ESAS

## Abstract

**Background**: Fractures of the radial head are common injuries, whereas, in the case of displaced fractures, surgical treatment using screw or plate osteosynthesis, excision, or replacement of the radial head is required. However, data about patient-related outcomes (PROM) for different types of radial head fractures is limited in the current literature. Therefore, this study was conducted to evaluate the functional outcome after operatively treated radial head fractures and to further correlate these results with the initial modified Mason classification. **Methods**: In this retrospective study, all suitable patients with surgical treatment of a radial head fracture were identified. Only patients with Mason II-IV fractures were included. All patients completed the Elbow Self-Assessment Score (ESAS) questionnaire. Data on fracture classification, type of surgery, and revision operations (if needed) were assessed. **Results**: A total of 92 patients suffering from fractures of the radial head (57 Mason II, 35 Mason III-fractures) who were operatively treated at our institution were enrolled. There were 42 (47.7%) female and 50 (54.3%) male patients with an average age of 47.5 ± 14.1 years. Screw osteosynthesis was performed in 67 patients, plate osteosynthesis in 20 patients, and five patients received radial head arthroplasty. The average ESAS score accounted for 89.7 ± 16.7. Mason II fractures showed significantly better functional results with higher ESAS scores (92.3 ± 13.9 vs. 85.4 ± 20.1) as well as significantly lower rates of necessary implant removal (0 vs. 5 (14.3%) than Mason III fractures. Screw osteosynthesis showed significantly better functional ESAS scores, 91.0 ± 16.5, than plate osteosynthesis, with 85.3 ± 17.6 (*p* = 0.041), but was predominantly used in Mason II fractures. **Conclusions**: Surgical treatment using screw- and plate osteosynthesis of radial head fractures provides a good overall outcome. The postoperative function is associated with the initial Mason classification as the patients’ reported outcome was worse in Mason III fractures compared to Mason type II fractures. In this context, the ESAS score can be considered a useful tool for the assessment of the patient-based functional outcome.

## 1. Introduction

Fractures of the radial head are common injuries with an incidence of 12.4/100,000 people [1], typically occurring at an average age of 48 years. Female patients are, on average, 10 years younger than the male cohort [2]. A fall on the outstretched hand with the elbow in a slightly flexed and pronated position presents the typical trauma mechanism. However, radial head fractures also appear as part of complex dislocation injuries of the elbow [3].

The most established classification system was originally published by Mason in 1954 [4]. Today’s most commonly used classification is the modified Mason classification described by Hotchkiss [5]. In addition, a treatment algorithm is derived from this modified classification. Mason type I fractures show minimal displacement (<2 mm) and mostly present with an undiminished range of motion. In these cases, conservative treatment is broadly accepted. Conservatively treated fractures of the radial head with a short immobilization of only 48 h provide good clinical results [6]. Fractures with a displacement of 2 mm or more without signs of comminution are classified as type II fractures. In these cases, motion may be mechanically blocked. The best treatment regime for Mason type II fractures is still controversially discussed. If surgery is decided upon, a screw osteosynthesis is usually performed. However, conservative therapy for Mason II fractures also provides comparable results, although the rate of postoperative radiological signs of osteoarthritis is higher [7]. Multiple fragments, along with severe comminution is considered as a type III fracture, which can only be treated by excision according to Hotchkiss’ publication in 1997 [5].

Over the years, surgical treatment options have improved, and not every comminuted radial head needs to undergo excision. Nonetheless, it is still common sense that Mason type III and IV fractures should be treated with open reduction and internal fixation (ORIF) using screw or plate osteosynthesis. Irreparable fractures can be treated by radial head prosthesis or resection of the radial head, depending on the extent of concomitant ligamentous injuries [8].

The overall outcome after radial head fractures shows good results not only for conservatively treated Mason I fractures [9] but also for complex Mason III types treated with radial head resection, prosthesis, or reconstruction [10,11]. However, the number of studies evaluating the functional outcome following radial head fractures is limited. In particular, not enough attention has been paid to patient-reported outcomes, which are nowadays considered an increasingly important benchmark [9,12]. Most studies compare the results between different treatment options for one fracture type (Mason classification), but different fracture types, according to Mason, were not compared.

For this reason, the aim of this study was to evaluate and compare the patient-reported functional outcome of surgically treated Mason type II and type III radial head fractures. As a hypothesis, it can be assumed that the surgical treatment of radius head fractures provides good results overall.

Mason type II fractures are probably associated with better functional outcomes than Mason III fractures. In addition, a correlation between the necessary treatment method (plate or screws) and the outcome is to be expected.

## 2. Materials and Methods

### 2.1. Study Population and Data Collection

In this single-center cohort study, the in-house fracture register was investigated for patients suffering from radial head fractures treated surgically between the years 2003 and 2016. Only isolated radial head fractures were included in the study. Patients suffering from complex injuries, including concomitant ligamentous injuries such as monteggia-like lesions or elbow dislocations, were excluded. Ligament stability is tested intraoperatively after the completion of the osteosynthesis. The width of the joint space under varus and valgus stress is assessed under fluoroscopic control. In the event of instability, this was addressed using reconstruction with suture anchors. Furthermore, multiple trauma was also considered an exclusion criterion.

### 2.2. Data Collection (Parameters)

Epidemiologic baseline information (age, gender) of enrolled patients was obtained from the hospital’s data management program (SAP SE, Walldorf, Germany). Based on the preoperative X-rays and CT scans, all fractures were classified according to the Hotchkiss modification of the Mason classification system. Whenever the Mason classification is mentioned in this paper, it refers to the modification by Hotchkiss [5]. Further information on follow-up operations, such as surgery for revision or elective implant removal, was assessed. In case the patient gave his informed consent, they were asked to complete the Elbow self-assessment score (ESAS) questionnaire.

The ESAS is a patient-reported outcome measure (PROM) that allows patients to rate their subjective elbow function [13]. The questionnaire was developed and validated by Beirer et al. in 2016. It includes illustrative photos, useful for the patient to determine the objective range of motion. Questions on pain level and functionality are also part of the ESAS. In addition to the subjective function, the questionnaire can also be used to objectively assess the range of motion in order to be able to determine a persisting postoperative flexion contracture of the elbow.

### 2.3. Surgical Technique

**Figure 1:**
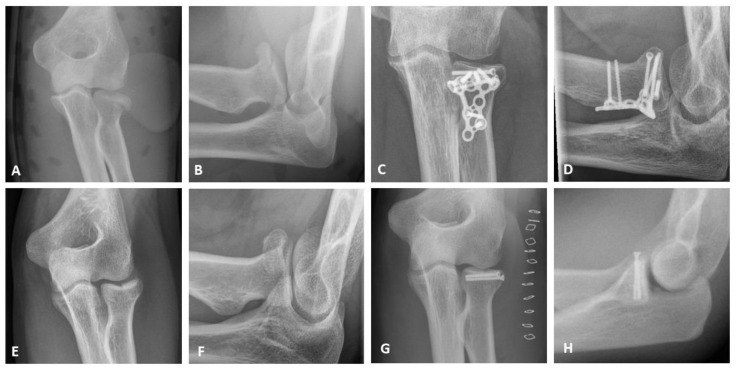
X-ray diagnostics; (**A**,**B**) Mason type II fracture in AP and lateral oblique view; (**C**,**D**) Mason type III fracture postoperatively after plate osteosynthesis in AP and lateral oblique view; (**E**,**F**) Mason type II fracture in AP and lateral oblique view; (**G**,**H**) Mason type II fracture postoperatively after screw osteosynthesis in AP and lateral view.

All patients underwent surgery via a lateral approach modified according to Kaplan [14]. The type of treatment was based on the instability and complexity of the fractures according to the Hotchkiss modification of the Mason classification. Simple 2-part fractures were stabilized using 2 mm lag screws (Medartis AG, Basel, Switzerland), whereas multi-fragmentary fractures were treated with 2.4 mm LCP radial head plates (DePuy Synthes GmbH, Oberdorf, Switzerland) as well as with 2.0 mm TriLock Radial Head Plates (Medartis AG, Basel, Switzerland). In case of irreparable fractures, radial head arthroplasty (RHA) was performed (MoPyC, Tornier SAS, Montbonnot Saint Martin, France). Figure 1 shows typical cases with pre- and postoperative x-rays of a mason type II fracture treated with screw osteosynthesis and a mason type III fracture treated with plate osteosynthesis.

### 2.4. Postoperative Treatment

The initial cast immobilization was terminated on day two after the surgery. Early functional exercising under physiotherapeutic assistance with a free range of motion was subsequently started. The prevention of postoperative stiffness of the elbow is essential in the aftercare of any type of radius head fracture. This applies to conservative therapy as well as to all surgical procedures such as screw osteosynthesis, plate osteosynthesis, and radial head prostheses. Therefore, all patients are instructed to start physiotherapeutic exercise at an early stage. Lifting weights was not allowed for six weeks until clinical and radiological control in our outpatient clinic took place.

### 2.5. Statistics

Frequencies of variables were specified with the number and the percentage share. For bivariate analyses, continuous variables were described using mean ± standard deviation. Binary variables were compared with percentages in cross-tables. Pearson’s-Chi-Square test was used to validate significance. Continuous variables were compared using the Student’s *t*-tests. Differences of not normally distributed variables were assessed using the Mann–Whitney-U Test. The level of significance was defined as *p* < 0.05. Statistics were calculated using SPSS (IBM SPSS Statistics for Windows, Version 22; Armonk, NY, USA).

## 3. Results

### 3.1. Baseline Epidemiological Data

**Figure 2:**
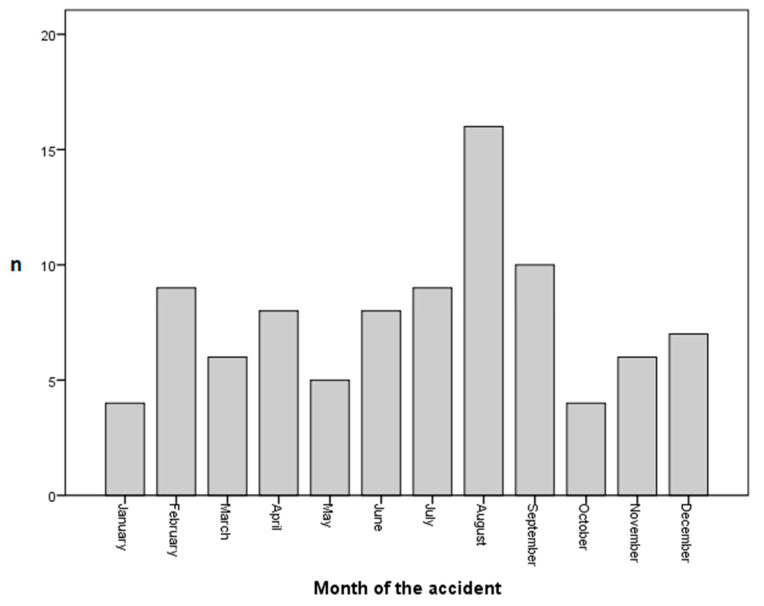
Monthly incidence of operatively treated radial head fractures.

A total of 92 patients met the inclusion criteria and returned the ESAS questionnaire: 42 (45.7%) of those patients were female and 50 (54.3%) were male. The average age was 47.5 ± 14.1 years, with a minimum of 17 years and a maximum of 77 years. The average time to follow up was 49.9 ± 37.1 months. Classification of the fracture morphology revealed 57 (62%) Mason type II fractures and 35 (38%) Mason type III fractures. The left elbow was affected more often (*n*= 49, 53.3%)) compared to the right side (*n*= 42, 45.7%). Analysis of the distribution of accidents over one calendar year showed a seasonal concentration in the summer months along with a peak in August (*n* = 16) (see Figure 2).

### 3.2. Treatment and Revisions

The majority of 67 (72.8%) fractures were treated using screw osteosynthesis, 20 (21.7%) patients underwent plate osteosynthesis, and for 5 (5.4%) patients the fracture was found to be irreparable, resulting in the implantation of a radial head prosthesis.

In three cases (3.3%), revisional surgery was indicated. Two patients suffered from secondary dislocation of the fragments after screw osteosynthesis. One patient suffered from persistent postoperative elbow instability, so a ligament reconstruction in terms of a revision was indicated as well. Elective implant removal was performed in 5 (5.4%) patients due to subjective irritation from the implant. Four (4.3%) of those implant removals were performed after plate osteosynthesis.

### 3.3. Outcome

Functional outcome was obtained using the ESAS PROM. The average functional outcome score reached 89.7 ± 16.7 points. There was no gender difference regarding the functional outcome. Female patients had an average ESAS Score of 89.7 ± 17.2 points, male patients of 89.7 ± 16.5 points. The patients’ age had no statistically relevant influence on the ESAS value (Pearson correlation coefficient −0.013 (*p* = 0.900).

Restriction of full extension of the elbow could be observed in 20 (20.7%) patients. Flexion was limited in 19 (20.7%) patients. Two (2.2%) patients reported a maximum flexion of 90° or less. Another 17 patients (18.5%) were able to bend to a maximum of 120°. All other 73 (79.3%) patients reported full ability of flexion, as shown in the elbow self-assessment questionnaire. Complications leading to revision surgery occurred in only 3 (3.3%) patients. Two patients who had been treated with screw osteosynthesis showed secondary dislocation of the fracture in the postoperative X-ray control. Therefore, revision with conversion to plate osteosynthesis was necessary in one of these patients and conversion to radial head prosthesis in the other. The third patient showed persistent ligamentous instability during the postoperative follow-up, so stabilization was carried out after 4 weeks using suture anchors.

### 3.4. Comparison of Mason Type II and III Fractures

**Figure 3:**
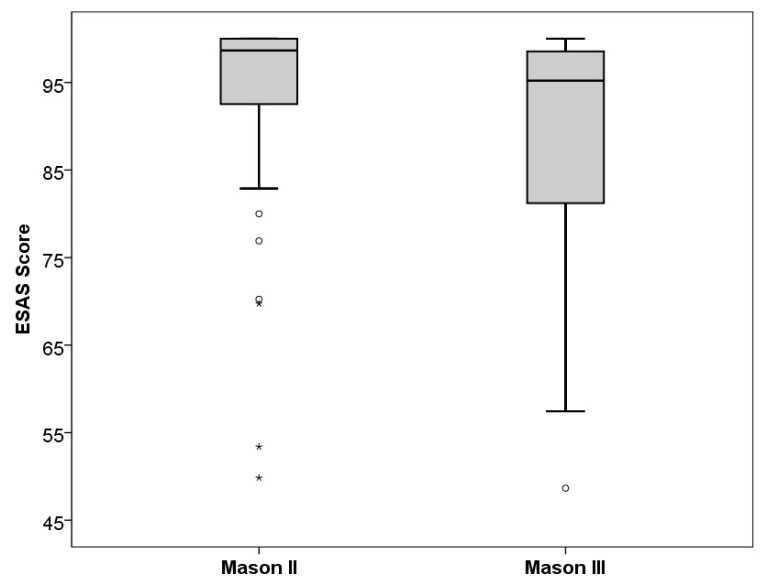
Boxplot diagram of ESAS scores in Mason type II and Mason type III fractures. (circles and stars mark outliers and extreme outliers).

Table 1 presents the differences between the Mason classification types. Looking at the epidemiological baseline data, no significant differences in age and gender were found. Since Mason III fractures are defined as unstable and presented with multiple fragments, these fractures were more likely to be treated using plate osteosynthesis. In this context, it should be mentioned that revision surgery and implant removal were only performed in cases of type III fractures. The difference in implant removal between Mason classification types reached significance (*p* = 0.007) but missed it regarding revision surgery (*p* = 0.052). The Elbow Self-Assessment Score was significantly lower for Mason type III fractures, indicating a poorer postoperative function (see Figure 3). This is also reflected in the range of motion, especially for the full extension of the elbow. Restricted range of motion could be observed in 9 (15.8%) cases of Mason type II fractures and 11 (34%) cases among type III fractures.

### 3.5. Screw vs. Plate Osteosynthesis

Besides the modified Mason classification, the type of ORIF was investigated in relation to the postoperative functional outcome. In total, 67 patients were treated with screw osteosynthesis, and 20 patients received plate osteosynthesis. Patients who received screw osteosynthesis showed significantly better functional outcome scores in the ESAS (91.0 ± 16.5) compared to patients treated with plate osteosynthesis (85.3 ± 17.6; *p* = 0.041). Furthermore, the rate of postoperative restricted ROM was significantly higher in the screw ORIF group (*n* = 10; 14.9%) compared to the plate ORIF group (*n* = 9; 45.0%) (*p* = 0.007). Implant removal was performed in 1.5% (*n* = 1) of patients treated with screws and in 20% (*n* = 4) of patients treated with plate osteosynthesis (*p* = 0.009).

Table 2 presents the analysis of functional outcomes in the group of Mason type III fractures. There was no difference between screw osteosynthesis and plate osteosynthesis in terms of functional outcome as measured using the ESAS score and the prevalence of restricted ROM. RHA, however, showed better functional ESAS scores without reaching the level of significance due to the small number of cases in the sample.

A multivariant linear regression analysis was performed using age, gender, fracture type according to the modified Mason classification, and type of surgery as variables, whereas the ESAS score was considered as the dependent outcome variable. This analysis did not reveal any of the mentioned variables as independent predictors, showing the following *p*-values: age *p* = 0.952; gender *p* = 0.898; Mason fracture type *p* = 0.071 and type of surgery *p* = 0.607.

## 4. Discussion

The presented study demonstrates a good overall postoperative functional outcome after operative treatment of Mason type II and III radial head fractures. Regardless of the type of operation, surgical therapy provides good results in the ESAS score with a low complication rate of only 3.3 percent.

There are several studies investigating the outcome after radial head fractures. Nevertheless, most studies only compare different treatment options for the same fracture type according to the Mason classification [7,11,15,16,17]. Sufficient data about the functional outcome comparing different Mason fracture types are still lacking in the current literature. The importance of patient-reported outcomes is constantly increasing in the evaluation process of postoperative outcomes. For this reason, this study was conducted to evaluate the overall postoperative outcome based on the patient-reported ESAS questionnaire in cases of displaced fractures of the radial head, including different types of fractures and treatment.

In his original publication describing the modification of the Mason classification, Hotchkiss also provided management guidelines, including a recommendation for treating Mason III fractures with excision or arthroplasty [5]. These recommendations can be repeatedly found in current literature [16,18,19,20]. In the presented sample of patients, only five cases were considered irreparable radial head fractures and, therefore, treated using RHA. All other enrolled fractures underwent surgical reconstruction using either screw or plate osteosynthesis showing good clinical results with an average ESAS score of 85.4 points in the Mason type III group. The continuous development in the field of implants with a special focus on anatomically preformed plates and interlocking screws for angular stability has led to a distinct improvement in the quality of radial head reconstructions so that arthroplasty or radial head resections are performed less frequently.

Despite the improvement in surgical methods and implants, Mason type III fractures remain challenging for upper extremity surgeons, usually resulting in poorer postoperative function. This is underlined by the results of this study, showing a lower average ESAS score of 85.4 ± 20.1 in the Mason III fracture group compared to an ESAS score of 92.3 ± 13.9 in the Mason II fracture group.

Although these facts may be self-evident to the experienced surgeon, there is no publication in the current literature that scientifically demonstrates this based on patient-reported outcome measures.

To the best of our knowledge, the presented study used the Elbow Self-Assessment score for the investigation of function after radial head fractures for the first time since its publication and validation in 2017 [13]. As of today, only a few studies have used the ESAS, which is why only a little evidence regarding the practical feasibility of this score exists [21,22]. The results of this study demonstrate that the ESAS can detect clinical differences in elbow functionality with high sensitivity. Furthermore, the quality of the clinical use of this PROM is confirmed using the presented results.

Lee et al. conducted a similar study on radial head fractures in 2018 [23]. Functional outcome and range of motion were investigated in a clinical examination by two Board-certified orthopedic surgeons using the QuickDASH score [24]. The authors found comparable results to this study with worse overall functional scores for Mason type III (QuickDASH 18 Range 0–68.2) compared to Mason type II fractures (QuickDASH 26.2 Range 0–86.4). This coherence with the QuickDASH score further demonstrates that the ESAS PROM is a useful tool for examiner-independent follow-up investigations of patients with elbow pathologies and is particularly suitable for use in register studies with high case numbers. The detection of the range of motion, which is not possible using other established scores like the DASH [24] or MEPS (Mayo Elbow Performance Score) [25], is an advantage of the ESAS.

Plate osteosynthesis provided worse functional results compared to screw osteosynthesis with regard to the ESAS score when the two procedures were compared independently of the fracture severity. Since plate osteosynthesis is used almost exclusively for Mason III fractures, it is only reasonable to look at this subgroup separately. Within the Mason III fracture group, no functional differences were found between plate and screw osteosynthesis. This suggests that the outcome is rather defined by the severity of the injury than by the type of surgery chosen. Nevertheless, the modified Mason classification could not be identified as an independent predictor of the functional outcome. Hereby, further investigations, including higher patient numbers, would be required.

Wu et al. also compared the outcome of screw-osteosynthesis with those of plate osteosynthesis, as well as with those of arthroplasty in 3-part radial head fractures and found functional outcomes comparable to our results but with a higher complication rate for plate osteosynthesis [15]. However, in their trial, no prospective, randomized study design was presented, so no reliable statements about the best treatment method can be derived.

In this study, Mason II fractures were very often treated surgically, which corresponds to the individual therapeutic algorithm of the authors. In general, however, there are still controversial opinions on the optimal treatment of isolated Mason type II radial head fractures. Guzzini et al. examined 50 patients with conservative treatment after mason type II fractures and described excellent functional outcome scores (DASH) and only marginal persistent limitations in range of motion [7].

In a systematic review of the literature, Lazerath et al. found that there were no differences in functional outcomes at mid-term follow-up when comparing Mason type II fractures treated surgically or conservatively. Nevertheless, the rate of osteoarthritis was higher in conservatively treated patients even though the mean follow-up period was shorter for the nonoperative cohort [26]. In our approach, we, therefore, favor surgical therapy for young patients to reconstruct the radiocapitellar joint surface as anatomically as possible, as the late effects of osteoarthritis are more evident in these patients.

The choice of the best possible osteosynthesis method always requires a precise preoperative analysis of the fracture. The final decision, however, depends on the intraoperative fracture assessment of the surgeon. A general statement regarding a superior osteosynthesis technique cannot be derived from the data.

### Limitations

This study is potentially subject to several biases. First, there is a possible selection bias between the patients who participated in the survey and those who declined to participate. Since the presented study is not a randomized trial, the choice of treatment was made by the surgeon. Therefore, no independent conclusion about the best surgical procedure can be derived from the present study results. Another limitation is the relatively low international application of the ESAS score. Even if the score is sufficiently validated, there are only limited studies that allow comparability.

## 5. Conclusions

This study demonstrates positive postoperative clinical outcomes of surgically treated radial head fractures with low rates of revision surgery. The higher complexity of Mason III fractures is reflected in slightly worse functional outcomes compared to Mason II fractures. Furthermore, this study shows good clinical applicability of the ESAS PROM whereby subjective function and gross range of motion can be measured in a valid way and independent of the examiner.

## Figures and Tables

**Table 1:** Baseline characteristics and outcome for Mason type II and type III fractures.

	Mason II	Mason III	*p*-Value
*n* (%)	57 (62%)	35 (38%)	
Age [years]	46.6 ± 11.9	49.1 ± 17.1	0.243
Gender female	27 (47.4%)	15 (42.9%)	0.419
*Surgery type*			<0.01 *
Screw osteosynthesis	56 (98.2%)	11 (31.4%)	
Plate osteosynthesis	1 (1.8%)	19 (54.3%)	
arthroplasty	0 (0%)	5 (14.3%)	
Flexion contracture	9 (15.8%)	11 (31.4%)	0.067
ESAS Score	92.3 ± 13.9	85.4 ± 20.1	0.022 *
Revision surgery	0 (0%)	3 (8.6%)	0.052
Implant removal	0 (0%)	5 (14.3%)	0.007 *

Data presented as mean ± SD or *n* (%); * = statistically significant.

**Table 2:** Functional outcome in Mason III fractures treated with different types of surgery.

	Surgery Type			
	Screw ORIF *n* = 11	Plate ORIF *n* = 19	RHA *n* = 5	*p*-Value
ESAS	84.6 ± 25.6	84.9 ± 17.9	89.1 ± 17.3 (18.2%)	0.887
Restricted ROM	2 (18.2%)	8 (42.1%)	1 (20%)	0.332

Data presented as mean ± SD or *n* (%); RHA = radial head arthroplasty; ORIF open reduction internal fixation.

## Data Availability

The data set can be made available on individual request to the corresponding author.

## Abbreviations

AP	anteroposterior
DASH	disabilities of the arm, shoulder and hand
ESAS	elbow self-assessment score
MEPS	mayo elbow performance score
ORIF	open reduction internal fixation
PROM	patient reported outcome measure
RHA	radial head arthroplasty
ROM	range of motion
